# Mast cell marker gene signature: prognosis and immunotherapy response prediction in lung adenocarcinoma through integrated scRNA-seq and bulk RNA-seq

**DOI:** 10.3389/fimmu.2023.1189520

**Published:** 2023-05-15

**Authors:** Pengpeng Zhang, Jianlan Liu, Shengbin Pei, Dan Wu, Jiaheng Xie, Jinhui Liu, Jun Li

**Affiliations:** ^1^ Department of Thoracic Surgery, The First Affiliated Hospital of Nanjing Medical University, Nanjing, China; ^2^ Department of Burns and Plastic Surgery, The First Affiliated Hospital of Nanjing Medical University, Nanjing, China; ^3^ Department of Breast Surgery, The First Affiliated Hospital of Nanjing Medical University, Nanjing, China; ^4^ Department of Rheumatology and Immunology, The Affiliated Drum Tower Hospital of Nanjing University Medical School, Nanjing, China; ^5^ Department of Gynecology, The First Affiliated Hospital of Nanjing Medical University, Nanjing, China

**Keywords:** lung adenocarcinoma, signature, mast cell, tumor immune microenvironment, SYAP1

## Abstract

**Background:**

Mast cells, comprising a crucial component of the tumor immune milieu, modulate neoplastic progression by secreting an array of pro- and antitumorigenic factors. Numerous extant studies have produced conflicting conclusions regarding the impact of mast cells on the prognosis of patients afflicted with lung adenocarcinoma (LUAD).

**Methods:**

Employing single-cell RNA sequencing (scRNA-seq) analysis, mast cell-specific marker genes in LUAD were ascertained. Subsequently, a mast cell-related genes (MRGs) signature was devised to stratify LUAD patients into high- and low-risk cohorts based on the median risk value. Further investigations were conducted to assess the influence of distinct risk categories on the tumor microenvironment. The prognostic import and capacity to prognosticate immunotherapy benefits of the MRGs signature were corroborated using four external cohorts. Ultimately, the functional roles of SYAP1 were validated through *in vitro* experimentation.

**Results:**

After scRNA-seq and bulk RNA-seq data analysis, we established a prognostic signature consisting of nine MRGs. This profile effectively distinguished favorable survival outcomes in both the training and validation cohorts. In addition, we identified the low-risk group as a population more effective for immunotherapy. In cellular experiments, we found that silencing SYAP1 significantly reduced the proliferation, invasion and migratory capacity of LUAD cells while increasing apoptosis.

**Conclusion:**

Our MRGs signature offers valuable insights into the involvement of mast cells in determining the prognosis of LUAD and may prove instrumental as a navigational aid for immunotherapy selection, as well as a predictor of immunotherapy response in LUAD patients.

## 1 Introduction

Lung cancer ranks among the most pervasive malignancies and constitutes the primary cause of cancer-related mortality, accounting for nearly two million new cases and 1.76 million fatalities annually (1). Lung cancer primarily bifurcates into small-cell lung cancer (SCLC) and non-small-cell lung cancer (NSCLC) (1). NSCLC, encompassing approximately 85% of all lung cancer incidents, emerges as the predominant variant, while LUAD represents the most ubiquitous histological subtype of NSCLC (2). LUAD is characterized by marked heterogeneity and an intricate tumor microenvironment (TME) (3). Conventional pathological staging falls short of fully prognosticating the outcomes for NSCLC patients, underscoring the need for devising novel and reliable prognostic models. Such models could facilitate the assessment of risk for LUAD patients, thereby informing the development of tailored immunotherapy and chemotherapy regimens.

TME was sculpted through the intricate interplay of neoplastic cells, immune cells, vasculature, extracellular matrix, stromal cells, fibroblasts, adipocytes, pericytes, and a multitude of signaling factors. Various cellular constituents within the TME engage in communication with tumor cells. Tumor-infiltrating immune cells, comprising the preponderance of the TME, interact with neoplastic cells, either promoting or inhibiting tumor growth, invasion, and metastasis (4). Mast cells, ubiquitously present across diverse tissues, assume a considerable role in the immune milieu of neoplastic tissues. These cells secrete pro- and anti-tumorigenic factors, modulating tumor development and dissemination. In recent years, tumor immunotherapy has emphasized the importance of immune cells within the TME as crucial targets. Mast cells are vital for altering the TME and facilitating tumor angiogenesis. Reports indicate that intratumoral mast cells exhibit enzyme profiles or subtypes distinctly different from their extratumoral counterparts (5–7). Within the TME, mast cells undergo activation and degranulation, adopting a highly proinflammatory state and actively recruiting macrophages and other innate immune cells to orchestrate the anti-tumor immune response. The role of mast cells in tumors remains a subject of debate, akin to macrophages, as inflammation-related mechanisms associated with mast cells may either foster or impede tumor formation (8). Due to the inflammatory mechanisms associated with mast cells, their role in tumor formation remains controversial, as is the case with macrophages (9). Research indicates that mast cells can modify their phenotype to exert various effects, and this transition can be regulated by macrophages and tumor cells (10). In gastric cancer, IL-33 secreted by tumor cells has been shown to activate mast cells and promote the expansion of tumor-associated macrophages (TAM). Patients with gastric cancer have been found to have poorer survival rates when TAMs accumulate (11). Investigations of colon cancer have revealed that mast cells can enhance the immunosuppressive properties of MDSCs through IFN production, and M2-type TAMs are significant producers of MDSCs (12). It has also been shown that tumor-associated mast cells are associated with overall and progression-free survival in lung cancer patients (13). In NSCLC, tumor-derived microvesicles (TMV) may activate mast cells to release cytokines and chemokines, affecting their migratory ability and potentially influencing tumorigenesis. The internalization of PKH67-labelled TMV from NSCLC cell lines by mast cells resulted in ERK phosphorylation, enhanced migratory ability, and increased release of cytokines and chemokines such as TNF-α and MCP-1 (14). Further research is needed to determine the potential therapeutic utility of mast cells in the treatment of LUAD.

By integrating single-cell and bulk RNA-seq data, we identified a gene signature comprising of nine MRGs that could potentially serve as therapeutic targets. Various analyses, such as survival analysis, TME, immune cell infiltration, and mutation analysis, were conducted to examine the underlying functions of the modeled genes. Furthermore, several cellular studies validated the role of SYAP1 in LUAD.

## 2 Methods

### 2.1 Dataset acquisition and processing

The scRNA-seq dataset GSE150938 contains 12 samples of lung adenocarcinoma (LUAD) obtained from the Gene Expression Omnibus (GEO) database (http://www.ncbi.nlm.nih.gov/geo/). Validation cohorts were obtained from the GSE31210 (n=226) and GSE30219 (n=85) datasets, while the GSE135222 (n=27) dataset was used for immunotherapy analysis validation. Clinical information, mutation data, and bulk sequencing data (n=516) for LUAD patients were acquired from The Cancer Genome Atlas (TCGA) database. The IMvigor210 dataset includes expression profiles and clinical data for patients with advanced uroepithelial carcinoma treated with immune checkpoint inhibitors (ICI). To improve comparability across datasets, all expression data were converted to transcripts per million (TPM) format and corrected for batch effects using the “batch” function of the “sva” package. Prior to analysis, all data were log-transformed.

### 2.2 Processing flow of scRNA-seq data

The scRNA-seq data underwent processing using the “Seurat” R packag (15). Genes expressed in fewer than 3 cells (min.cells = 3) and cells with fewer than 200 detected features (min.features = 200) were excluded. After filtering out cells with nFeature RNA > 7000 and mitochondrial sequencing count > 10%, a total of 46286 cells were retained for further analysis. The dataset was normalized using Seurat’s NormalizeData and ScaleData routines, and batch effect was eliminated using CCA. The top 2000 genes exhibiting the highest degree of variability were selected using the “FindVariableFeatures” algorithm. Clusters were identified using Seurat’s Stochastic Neighbor Embedding (tSNE) (16) and the “FindClusters” function (resolution = 0.8). Marker genes were identified using the “FindAllMarkers” function in Seurat, with cluster-specific markers being retained when log2FC exceeded 0.25. Cells were classified using common cell markers, and for further analysis, the differential genes in the mast cell cluster were treated as mast cell marker genes. The “AUCell” R package was used to determine the activity of a gene set in individual cells, with gene expression rankings of each cell being based on the area under the curve (AUC) value of the model genes to compute the fraction of highly expressed gene sets in each cell. AUC values were higher in cells with greater expression of the gene set, and the “AUCell exploreThresholds” function was utilized to calculate the threshold for identifying cells with active gene sets. The “ggplot2” R program was then employed to visualize the active clusters by translating each cell’s AUC score to a t-SNE embedding.

### 2.3 Building and evaluating prognostic models

The TCGA cohort’s overall survival (OS) was evaluated using a univariate Cox analysis (17), and MRGs with prognostic significance were identified based on a *P<*0.05. The “glmnet” package was employed to select alternative genes and construct prognostic features using the least absolute shrinkage and selection operator (LASSO) (18). The “caret” package segregated LUAD patients into two groups based on a 7:3 ratio for training and testing. The MRGs signature was generated using the following equation: $risk\;score=\sum_{k=0}^{n}Exp\;k*\;coef\;k$ . where Expi represents the expression level of each MRGs and coef k represents the corresponding model gene coefficient. The risk score for each patient in both the TCGA training and test groups was determined using the aforementioned method, and patients were classified as either high risk or low risk based on the median risk score. The signature was validated in the GEO cohorts, with the risk score calculated in a manner consistent with the TCGA dataset. The accuracy of the model was assessed using the ROC curve, and external validation of this signature was performed using the risk scores calculated using the aforementioned formula for survival analysis in LUAD samples from the GEO database.

### 2.4 Correlations among model genes and their impact on survival

The extraction of model genes’ expression was performed, followed by a correlation analysis to investigate the correlation and *P* value among the model genes and between the model genes and risk scores. The obtained results were presented using the “GGally” R package. To determine the contribution of each model gene to the OS, the “survcutpoint” function was employed, which computed optimal cutpoint values for each gene and produced corresponding survival curves. In addition, a heatmap was generated to examine the association between the risk model and clinical features in greater detail by incorporating clinical data, model genes, and immune checkpoint gene expression.

### 2.5 Nomogram construction

The clinical characteristics were integrated with the risk score utilizing the R package ‘rms’ to construct a more precise nomogram (19, 20), which enhanced the predictability of prognostication. The accuracy of the nomogram was assessed by calibration and ROC curves (21).

### 2.6 Enrichment analysis and functional annotation

The “limma” package was utilized to determine DEGs between high- and low-risk groups, with a threshold of FDR< 0.05 and log2 (FC) > 1. Gene set enrichment analyses (GSEA) and Gene Ontology (GO) enrichment were conducted for differential genes between different risk groups, with assistance from the R packages “clusterProfiler” and “org.Hs.eg.db” (22). In addition, Gene Set Variation Analysis (GSVA) was performed to investigate the heterogeneity of various biological processes, using the “GSVA” package. The Hallmark gene sets “h.all.v7.5.1.symbols.gmt” from MSigDB were utilized for the GSVA. Functional annotation was carried out using the R tool “clusterProfiler”. The enrichment scores of LUAD samples were determined using single sample gene set enrichment analysis (ssGSEA). It was observed that there were differences in pathway activity between the high-risk and low-risk groups, as indicated by the T values. To present the results of the enrichment analysis, the R packages “ggplot2” and “GseaVis” were employed.

### 2.7 Mutation analysis

The somatic mutations of LUAD in low- and high-risk groups were analyzed using the R package “maftools” and the mutation annotation format (MAF) was generated using TCGA database data (23). Tumor mutation burden (TMB) was estimated for each LUAD patient.

### 2.8 Immunotherapy and evaluation of immune microenvironment

In order to characterize the immune microenvironment, the CIBERSORT method with 1,000 permutations was used to determine the composition of 22 different types of immune cells (24). We acquired the LUAD patient data from the Timer2.0 database and used seven immune infiltration assessment methods to analyze all TCGA database patients. The differences in immune cell infiltration between different risk groups were examined, and the degree of immune cell infiltration was visualized using heat maps. Boxplots and scatterplots were used to display differences and correlations in immune checkpoint genes between high- and low-risk groups. Using single sample gene set enrichment analysis (ssGSEA), the enrichment scores of 29 immune signatures were quantified. Additionally, the R package “estimate” was utilized to determine the immunological scores, stromal scores, and ESTIMATE scores of LUAD patients (25, 26). The immunogenicity of LUAD was assessed using machine learning. Immunophenoscores (IPS) were retrieved from the Cancer Immunome Atlas (TCIA) database for LUAD (27). The IPS scores between the high-risk and low-risk groups were compared to predict immunotherapy sensitivity. To evaluate the likelihood of response to ICI treatment, the Tumor Immune Dysfunction and Exclusion (TIDE) algorithm was utilized online (http://tide.dfci.harvard.edu/) (28). Lower TIDE scores indicated a greater probability of responding to ICI treatment with greater effects. Finally, two immunotherapy cohorts, IMvigor210 and GSE135222, were used to assess the model’s ability to predict immunotherapy outcomes.

### 2.9 Relationship between risk model and six immune subtypes and pathological stage

There exist six established immune subtypes, namely wound healing, inflammatory, lymphocyte deficient, immunologically quiet, and TGF-dominant (29). To assess these subtypes in LUAD samples and compare them to the constructed risk model, the “ImmuneSubtypeClassifier” software was employed. The differences were analyzed using the Chi-square test. Similarly, the association between the high- and low-risk groups and pathological stage was assessed using the same method.

### 2.10 Cell lines culture

Normal human lung epithelial BEAS-2B cells and human LUAD cell lines (A549, H1299) were procured from the Cell Resource Center of Shanghai Life Sciences Institute and cultivated in F12K or RPMI-1640 (Gibco BRL, USA) supplemented with 10% fetal bovine serum (FBS) and 1% streptomycin and penicillin (Gibco, Invitrogen, Waltham, MA, USA) under 5% CO2, 95% humidity, and 37°C conditions.

### 2.11 Cell transfection

Small interfering RNAs (siRNAs) constructs (GenePharma, Suzhou, China) were utilized to generate SYAP1 knockdown (30). **Supplementary Table S1** lists the SYAP1 siRNA sequences. Cells were seeded in a 6-well plate at 50% confluence and transfected with negative control (NC) and knockdown (siSYAP1) using Lipofectamine 3000 (Invitrogen, USA).

### 2.12 Extraction of RNA and Real-Time PCR (RT-PCR)

Total RNA was extracted from cell lines using TRIzol (15596018, Thermo) following the manufacturer’s instructions, and cDNA was synthesized using the PrimeScriptT-MRT kit (R232-01, Vazyme). Real-time polymerase chain reaction (RT-PCR) was per-formed using SYBR Green Master Mix (Q111-02, Vazyme), with mRNA expression lev-els being normalized to the level of GAPDH mRNA. The expression levels were calcu-lated using the 2−ΔΔCt method, and all primers were obtained from Tsingke Biotech (Beijing, China). **Supplementary Table S1** contains the full primer sequences.

### 2.13 Cell Counting Kit-8 experiment (CCK-8)

1×103 cells were transfected into each well of a 6-well plate and cultured for 14 days. The cells were washed twice with PBS and fixed with 4% paraformaldehyde for 15 minutes before being stained with Crystal Violet (Solarbio, China).

### 2.14 Colony formation

We transfected 1×10^3^ cells into each well of a 6-well plate and kept the cells alive for 14 days. Before Crystal violet (Solarbio, China) staining, the cells were washed twice with PBS and fixed for 15 minutes in 4% paraformaldehyde.

### 2.15 EdU

For the EdU assay, a 96-well plate containing 2×10^4^ treated cells per well was uti-lized. The cells were allowed to attach to the well before conducting the assay following the manufacturer’s recommended protocol (Ribobio, China) for 5-Ethynyl-2’-deoxyuridine (EdU) incorporation. The number of proliferating cells was counted using an inverted microscope.

### 2.16 Wound-healing assay

Transfected cells were plated in 6-well plates and incubated until they reached 95% confluency. Using a sterile 20-L plastic pipette tip, a single straight line was scraped in each cultured well, and unattached cells and debris were washed away twice with PBS. The width of the scratch wounds was measured using the Image J software by taking photos of the wounds at 0 and 48 hours.

### 2.17 Transwell assay

For the cell invasion and migration assays, transwells were used. Treated A549 and H1299 cells (2×105) were added to the top chamber of 24-well transwells and incubated for 48 hours. The top section of the plate was either precoated with matrigel solution (BD Biosciences, USA) or left untreated to evaluate the cells’ invasion and migration ability. The cells on the top surface were removed, and the remaining cells on the bottom layer were fixed with 4% paraformaldehyde and stained with 0.1% crystal violet (So-larbio, China).

### 2.18 Analysis of apoptosis

To induce apoptosis, all cells were exposed to 0.5 mM H2O2 for 4 hours before apoptosis analysis. An Annexin V-APC/PI Apoptosis Detection Kit (KeyGEN, Jiangsu, China) was used following the manufacturer’s instructions. The apoptotic rate was then determined using the BD FACSCanto II (BD Biosciences, San Jose, CA, USA).

## 3 Results

### 3.1 Identification of mast cell markers

**Figure 1:**
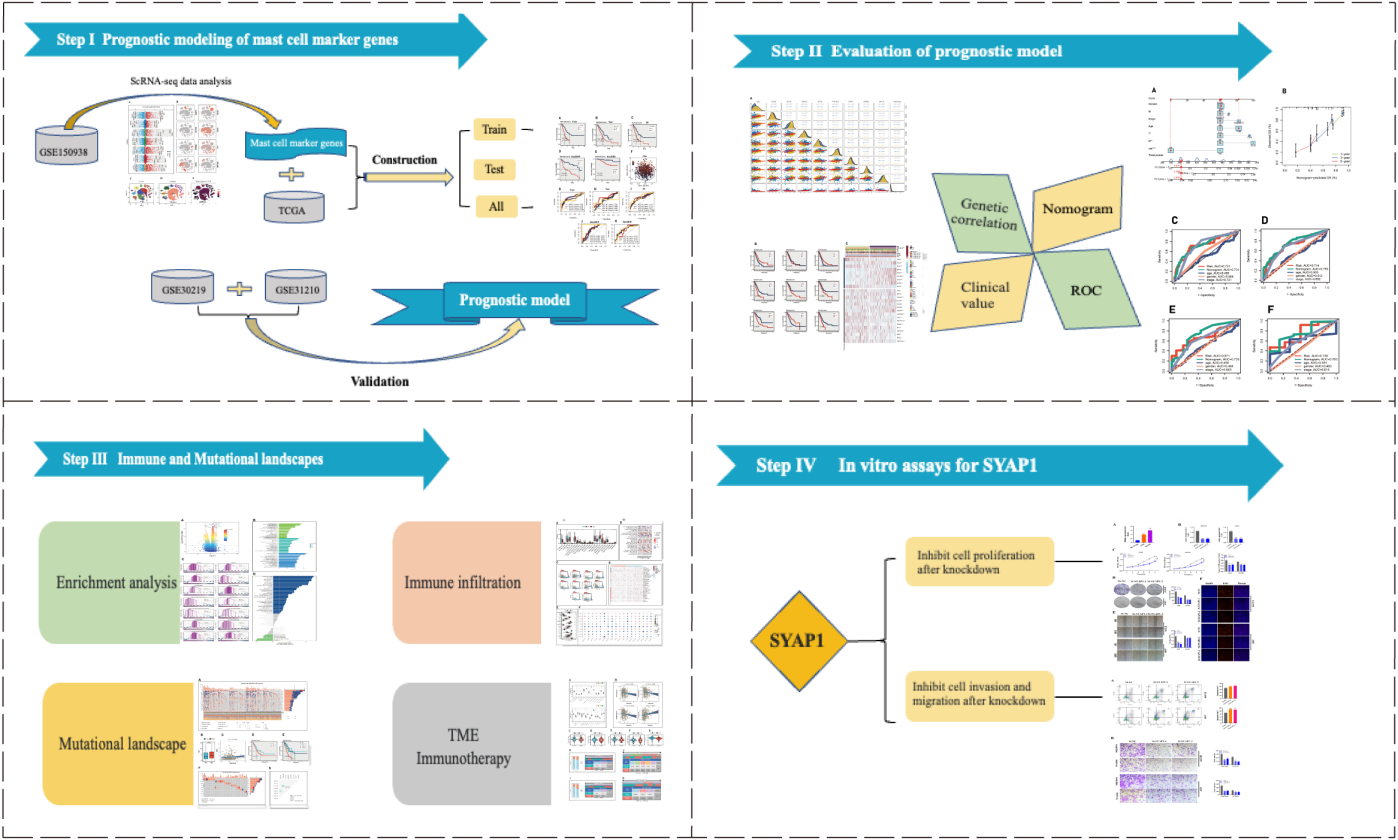
The flowchart of this investigation. In total, we divided this study into four parts, construction of the prognostic model; assessment of model stability; application of the model in predicting immunotherapy; cancer-promoting effect of SYAP1 in LUAD cells.

**Figure 2:**
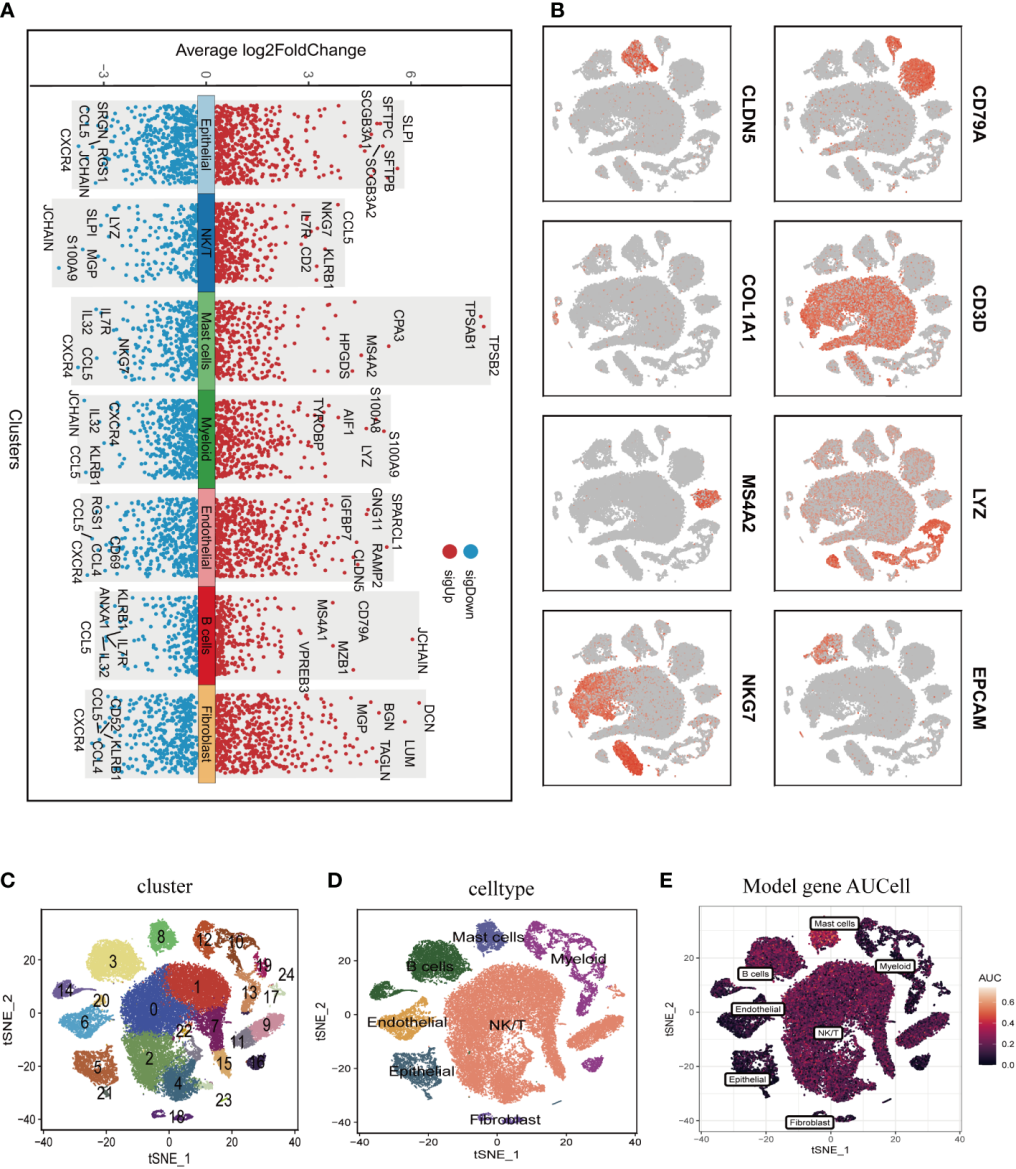
Annotation of single-cell data and extraction of Mast cell. **(A)** Differentially expressed marker genes were used to identify each cell group. **(B)** Typical marker genes for each cell group. **(C)** The tSNE plot showed that all the cells in the 12 lung adenocarcinoma samples can be classified into 24 clusters. **(D)** The tSNE map indicates that lung adenocarcinoma samples can be annotated as 7 cell types in the tumor microenvironment (different colors represent different cell types). **(E)** AUCell score of model genes in each cell.

**Figure 1** illustrated the study’s schematic representation, while **Supplementary Figure 1** provided comprehensive quality control information for scRNA-seq analysis. After data processing and filtering, gene expression profiles for 46,286 cells derived from 12 LUAD samples were acquired and subjected to further investigation. Marker genes exhibiting differential expression were employed to distinguish each cellular group, as depicted in **Figure 2A**, and representative marker genes for each group were displayed in **Figure 2B**. Following dimensionality reduction and log-normalization, 24 cell clusters were generated (**Figure 2C**). Annotations for cell identification within each cluster were determined by comparing DEGs to canonical marker genes. Cells in cluster 8 were identified as mast cells (**Figure 2D**), resulting in the detection of 306 LUAD mast cell gene markers. Upon mapping these marker genes to TCGA and GEO databases, 294 genes were preserved. Furthermore, **Figure 2E** demonstrated that AUCell scores of model genes were notably elevated in mast cells.

### 3.2 Building a predictive signature

**Figure 3:**
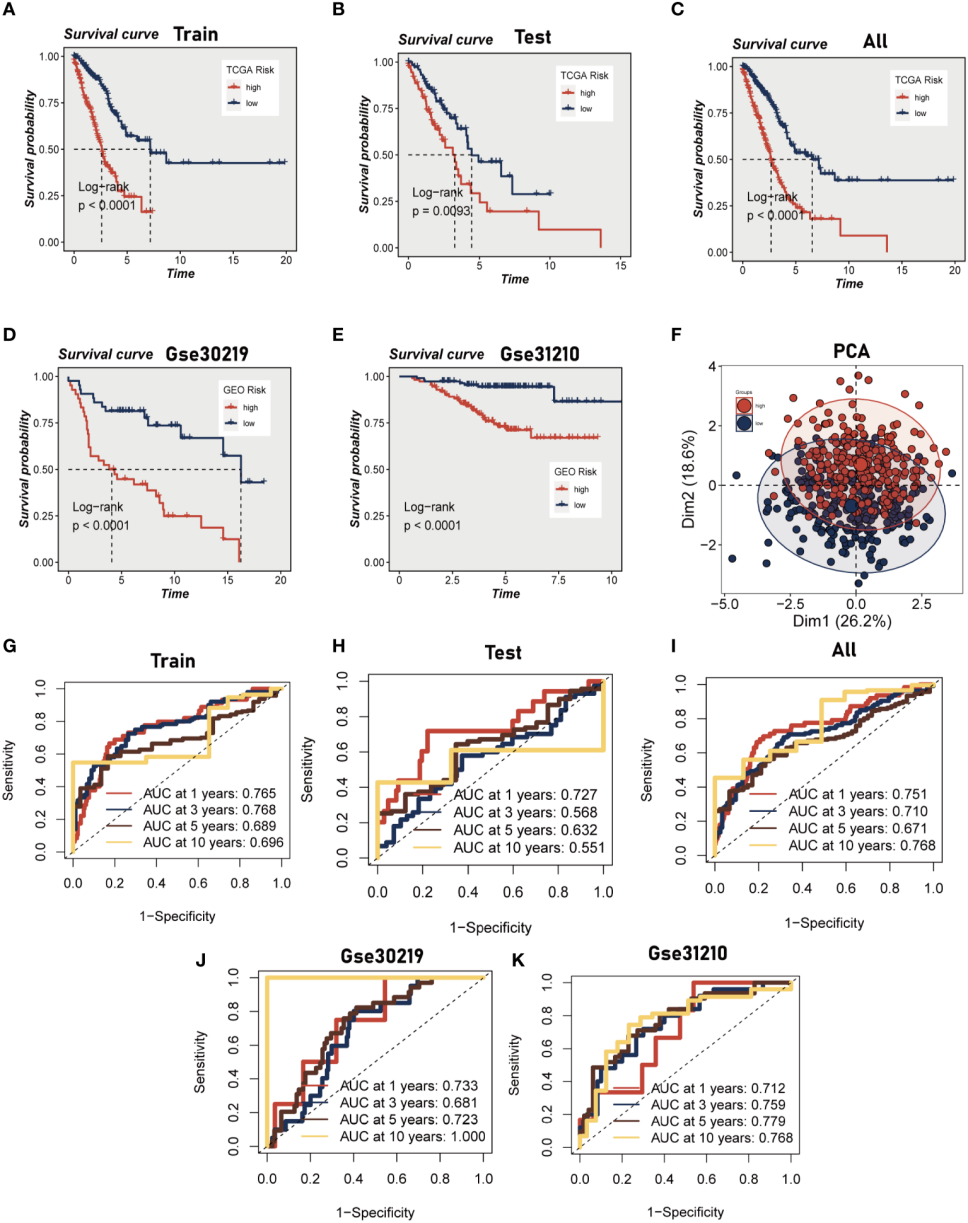
Construction and validation of Mast cell-related prognostic model. **(A–E)** Kaplan-Meier prognostic analysis of signatures in the training, testing, whole TCGA, GSE31210 dataset and GSE30219 dataset. **(F)** PCA analysis in TCGA cohort. It was found that the model could group LUAD patients well. **(G–K)** Time-dependent ROC curves of signatures in the training, testing, whole TCGA, GSE31210 dataset and GSE30219 dataset.

Initially, we employed a univariate Cox proportional regression analysis on the TCGA training dataset to identify 50 mast cell marker genes that exhibited statistically significant associations with OS (*P*<0.05, **Supplementary Figure 2**). These genes were then utilized to construct a prognostic signature. The most advantageous prognostic genes were determined through a LASSO Cox regression model, selecting one standard error above the minimum criterion, which resulted in a 20-gene model comprising RGS13, HDC, RAB27B, RGS2, BEX4, NFKBID, BIRC3, ELL2, CNIH1, AP3S1, EIF3E, LAT, DDIT4, DNAJB4, PPP3CA, SYAP1, AP2M1, EIF2S3, BANF1, and MYLIP. A multivariate Cox regression analysis was employed to pinpoint a final set of nine genes, which were subsequently used to refine the model to encompass only the most predictive genes. A risk score was subsequently generated: risk score = (-0.28914 × HDC expression) + (0.146946 × RGS2 expression) + (-0.249 × BEX4 expression) + (0.204576 × BIRC3 expression) + (0.391839 × EIF3E expression) + (0.272907 × PPP3CA expression) + (0.402948 × SYAP1 expression) + (0.363513 × AP2M1 expression) + (-0.3329 × MYLIP expression). The same algorithm was applied to the TCGA validation set, the TCGA all set, and two additional independent GEO datasets. Patients were stratified into high- and low-risk categories based on the median risk score. Kaplan-Meier survival analysis indicated that high-risk patients experienced significantly poorer OS compared to low-risk patients (*P*<0.01, **Figures 3A–E**). Principal component analysis (PCA) demonstrated that the risk score could effectively segregate the TCGA data into two distinct groups (**Figure 3F**). Utilizing ROC curves for OS at 1, 3, 5, and 10 years, we assessed the prognostic accuracy of the prognostic signature. In the TCGA lung adenocarcinoma (LUAD) training cohort, the area under the curve (AUC) values at these time points were 0.76, 0.76, 0.68, and 0.69, respectively (**Figure 3G**). In the TCGA validation set and the entire set, superior AUC values were also exhibited (**Figures 3H, I**). Analogously, the AUC values of the two additional GEO cohorts indicate that the model possesses a commendable degree of accuracy. For the GSE30219 cohort, the AUC values at 1, 3, 5, and 10 years are 0.712, 0.759, 0.779, and 0.768, respectively, while for the GSE 31210 cohort, they are 0.733, 0.681, 0.723, and 1.000 (**Figures 3J, K**).

### 3.3 Correlations between model genes and impact on survival

**Figure 4:**
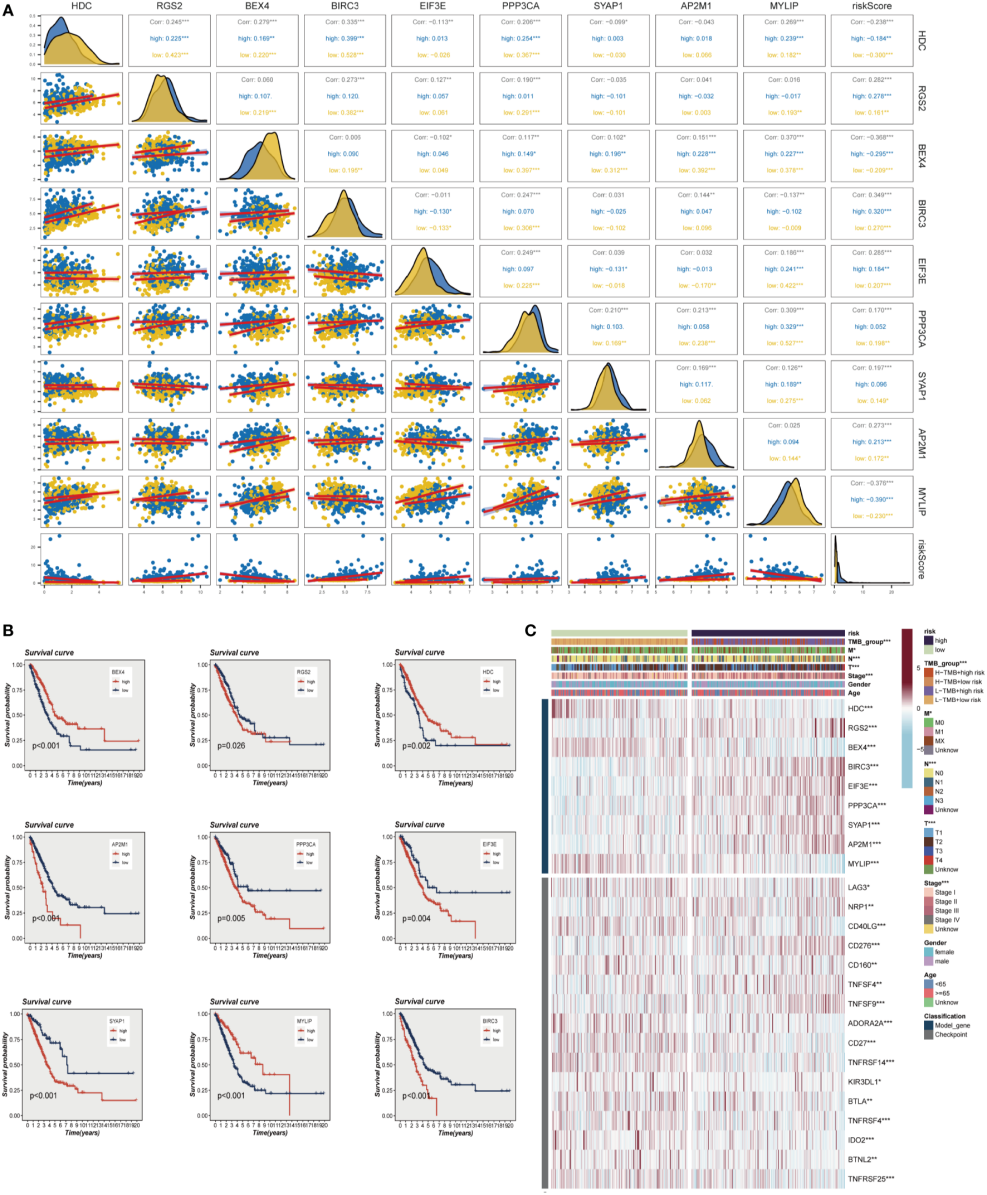
Correlations between model genes and their impact on survival. **(A)** Correlation analysis between model genes and risk scores. **(B)** The relationship between model genes and survival. **(C)** A heat map incorporating clinical data, model genes, and immune checkpoint gene expression. *P < 0.05, **P < 0.01, ***P < 0.001.

The evaluation of the expression levels of model genes revealed that the majority of genes exhibited positive correlations among their expression levels, with BEX4 and PPP3CA demonstrating the most positive correlations with MYLIP (R>0.3, *P*<0.001). The marginally negative relationship between BEX4 and BIRC3 and EIF3E was not statistically significant. We also observed that the genes and model risk scores were closely associated, with HDC, BEX4, and MYLIP displaying considerable negative correlations, while the remaining genes exhibited significant positive correlations (**Figure 4A**). The impact of each gene on survival is depicted in **Figure 4B**, which indicates that HDC, BEX4, and MYLIP serve as protective genes, suggesting that reducing the expression of these genes may enhance patients’ OS. Conversely, RGS2, BIRC3, EIF3E, PPP3CA, SYAP1, and AP2M1 are high-risk genes, and their elevated expression may diminish patient survival. **Figure 4C** demonstrates that the high-risk group exhibits increased expression of RGS2, BIRC3, EIF3E, PPP3CA, SYAP1, and AP2M1, whereas the low-risk group displays heightened expression of other model genes. Additionally, the graph also highlights significant differences between the high-risk and low-risk groups in terms of the expression of certain immune checkpoint genes, such as TNFRSF25, BTNL2, IDO2, and TNFRSF4, among others.

### 3.4 Nomogram construction

**Figure 5:**
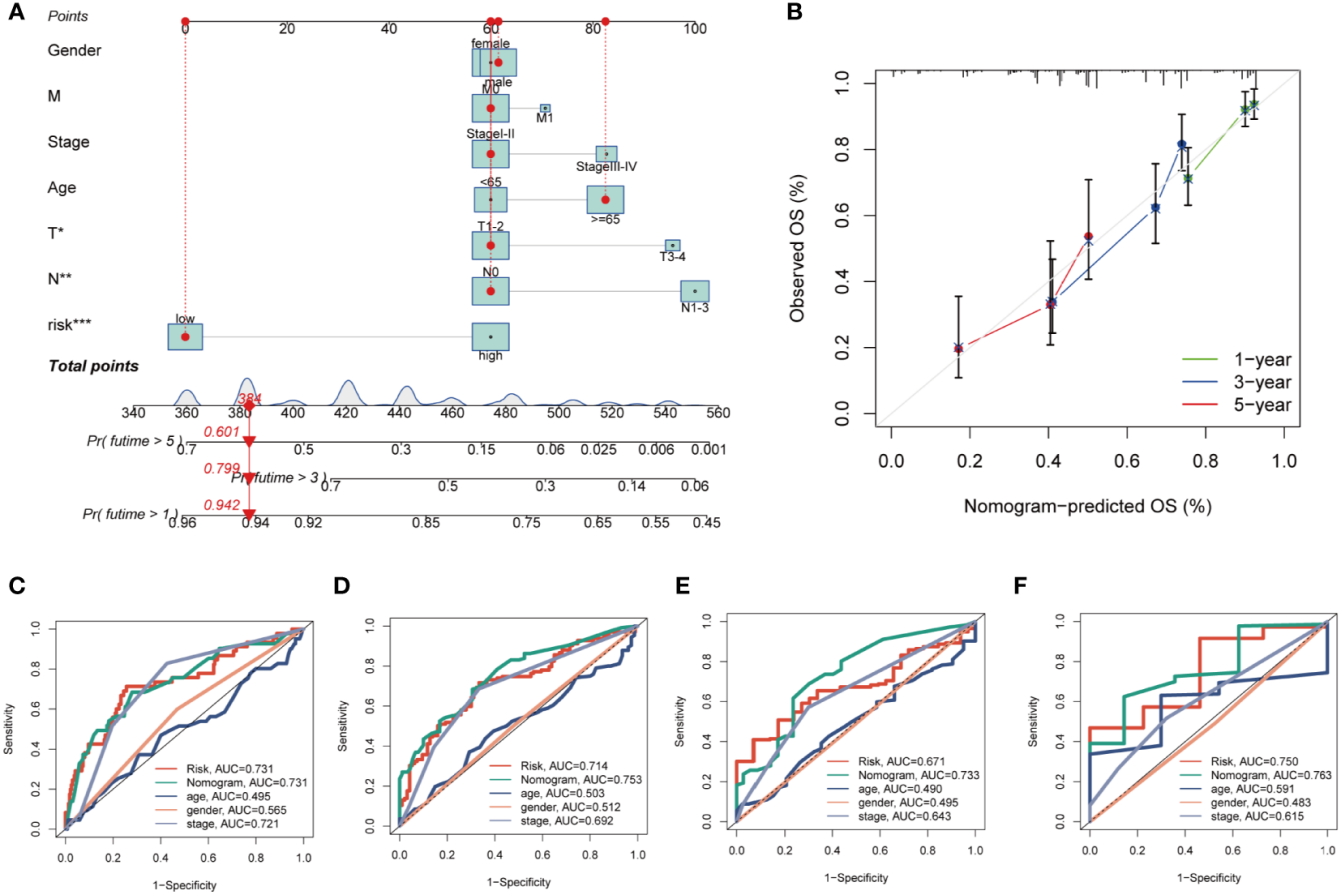
Building a more accurate nomogram. **(A)** Nomogram was constructed by combining Clinical features with risk groups. **(B)** Nomogram’s 1-, 3, and 5-years calibration curve. **(C–F)** ROC curves for 1, 3, 5 and 10 years showed AUC values for various clinical factors, risk scores, and nomogram scores. *P < 0.05, **P < 0.01, ***P < 0.001.

By integrating clinical data and risk stratification, a nomogram was devised to assess the risk of TCGA patients. **Figure 5A** illustrates the patients’ gender, age, T, N, M stages, overall stage, and risk classification. This nomogram may facilitate a more precise determination of patient risk and guide subsequent therapeutic strategies. To further appraise the accuracy of this nomogram, prognostic ROC analysis was conducted, revealing that the findings notably surpassed those of alternative clinical models and risk scores. According to the results, the AUC values at 1, 3, 5, and 10 years were 0.731, 0.753, 0.733, and 0.763, respectively (**Figures 5C–F**). Additionally, we generated calibration curves (**Figure 5B**) and noted that this nomogram could reliably forecast the prognosis of LUAD patients at one, three, and five years.

### 3.5 Analysis of enrichment

**Figure 6:**
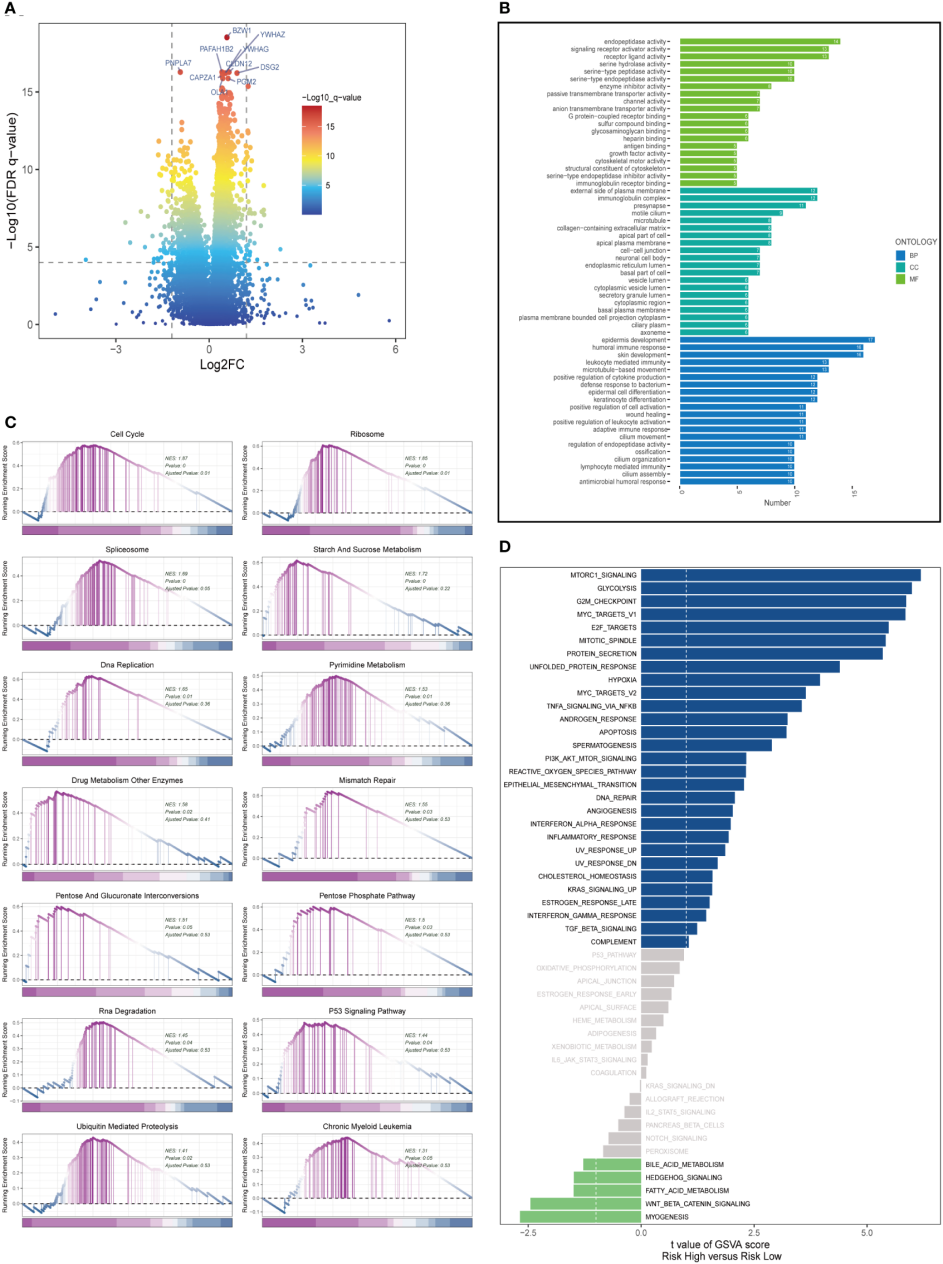
Enrichment analysis and functional annotation. **(A)** Differentially expressed genes between high- and low-risk groups (FDR <0.05, log2 (FC) > 1). **(B)** Bar graphs are used to show GO enrichment analysis. **(C)** GSEA enrichment method showed pathway differences between high and low risk groups. **(D)** GSVA shows the enrichment of hallmark gene sets in different risk groups.

In both high- and low-risk cohorts, DEGs were identified and their expression profiles were visualized using a volcano plot (FDR< 0.05 and log2 (FC) > 1; see **Figure 6A**). Gene Ontology (GO) enrichment analysis was performed on the DEGs (**Figure 6B**) and revealed that the biological processes (BP) mainly involved skin development, humoral immune response, and epidermis formation. In terms of cellular component (CC), the immunoglobulin complex, presynapse, and external side of the plasma membrane were the most represented. In molecular function (MF), endopeptidase activity, receptor ligand activity, and signaling receptor activator activity were the predominant categories. It has been reported that the activity of prolyl endopeptidase is associated with the prognosis of colorectal cancer (31). GSEA was conducted on high and low-risk LUAD groups (**Figure 6C**). GSEA enrichment analysis revealed that cell cycle (NES = 1.87, p = 0.000), ribosome (NES = 1.85, p = 0.000), spliceosome (NES = 1.69, p = 0.000), starch and sucrose metabolism (NES = 1.72, p = 0.000), DNA replication (NES = 1.65, p = 0.000), pyrimidine metabolism (NES = 1.53, p = 0.01), drug metabolism other enzymes (NES = 1.58, p = 0.02), mismatch repair (NES = 1.5, p = 0.03), and others were enriched in LUAD patients with high-risk scores. Analysis of hallmark pathway gene signatures underscored that the high and low-risk groups exhibited some distinctions. A direct comparison of Risk-High versus Risk-Low revealed the top 5 enriched signatures in the high-risk group included mTORC1 signaling, Glycolysis, G2M checkpoint, C-MYC target, and E2F targets (**Figure 6D**). Prior studies have demonstrated that, despite oxygen availability, glycolysis is augmented in numerous malignancies; however, therapeutic investigations using glycolysis inhibitors, such as the glucose analog 2-deoxy-D-glucose (2DG), have proven ineffective. MTORC1 signaling instigates metabolic reconfiguration, allowing tumor cells to become glycolysis-independent, thus promoting the initiation and progression of malignancies. Furthermore, c-Myc is essential for tumorigenesis (32), Myc frequently enhances transcription (33), indicating that LUAD cells may be susceptible to Myc inhibition. The E2F family encodes crucial nuclear transcription factors involved in regulating the cell cycle (34, 35). Clinical research suggests that E2F family members are directly associated with the incidence, progression, proliferation, and apoptosis of various cancerous tumors such as gastric, lung, liver, esophageal, prostate, bladder, and ovarian cancer (34, 36). The G2M checkpoint also operates as a cell cycle regulatory pathway to control cell proliferation. Elevated G2M checkpoint pathway activation has been linked to significantly poorer survival in pancreatic cancer patients (37). Consequently, these pathways, more prevalent in the high-risk group, may play a vital role in regulating tumor growth in LUAD.

### 3.6 Association between mutation and prognosis

**Figure 7:**
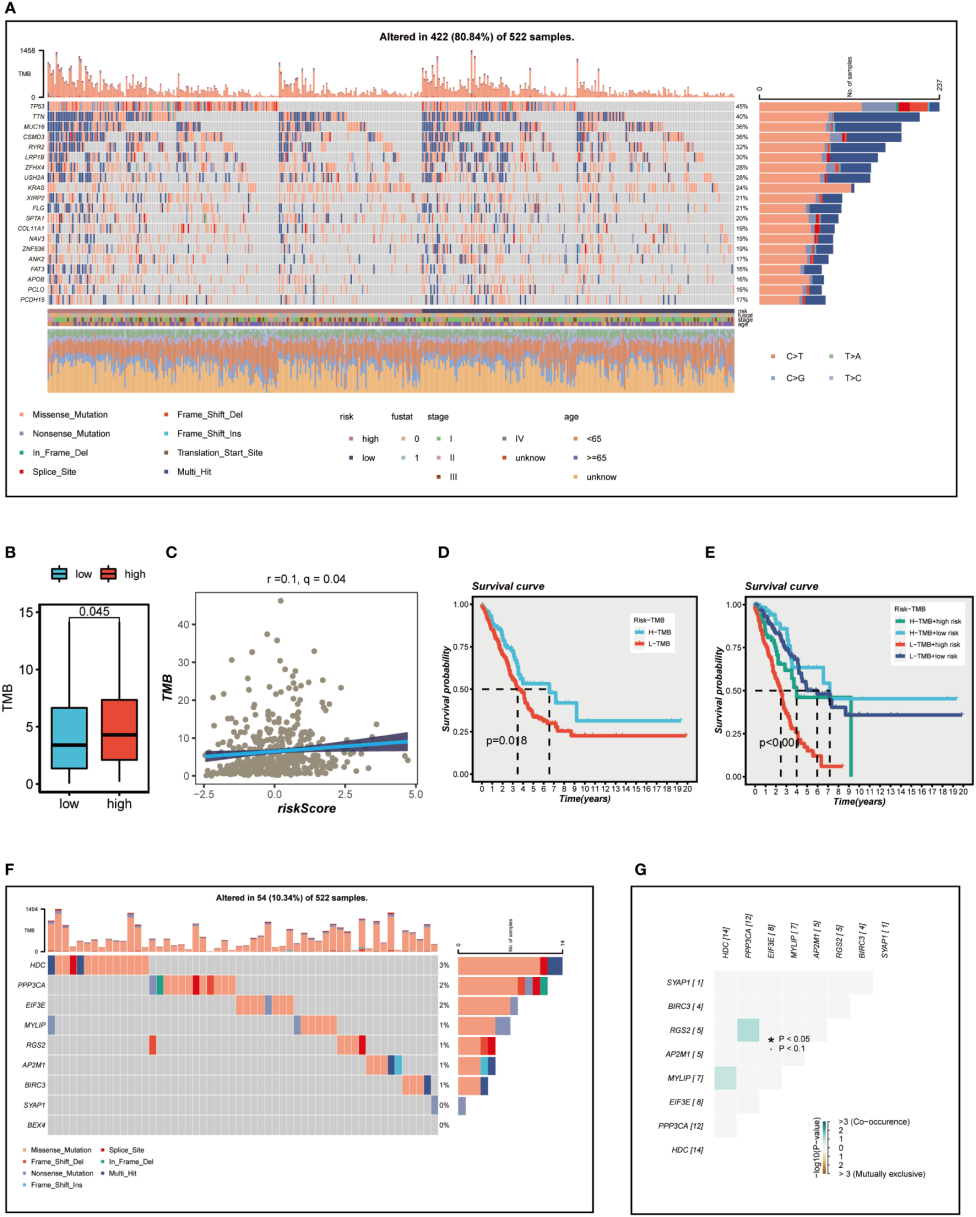
Mutation analysis. **(A)** Mutation landscape of the top 20 genes with mutation frequency in the high-low risk group. **(B)** TMB differences between high - and low-risk patients. **(C)** Relationship between risk score and tumor mutation burden. **(D)** Survival differences between patients with high and low TMB. **(E)** Survival analysis between four different groups (H-TMB+high-risk, H-TMB+low-risk, L-TMB+high-risk, and L-TMB+low-risk). **(F)** Mutation landscape of model genes. **(G)** Co-mutation or co-exclusion relationships among model genes. *P < 0.05.

**Figure 7A** presents an overview of the mutations discovered in LUAD specimens, with missense mutations being the most common type. In comparison to other single nucleotide locus variation (SNV) classes, C>T exhibited the highest frequency. The top 20 genes with the highest mutation frequency in the high and low-risk groups are displayed. It is evident that the top five genes (TP53, TTN, MUC16, CSMD3, RYR2) with the highest mutation frequency in the high-risk group demonstrate distinct levels of increased mutation samples compared to those in the low-risk group. We subsequently examined the differences in TMB between the high- and low-risk groups (**Figure 7B**) and conducted a correlation analysis (**Figure 7C**). The results indicated a significant positive association between TMB and risk score, as well as notably different TMB levels between the two risk groups. We then investigated how the two groups, in conjunction with TMB, could influence OS prognosis. Survival analysis suggested that higher TMB levels were associated with improved OS (**Figure 7D**). Based on the median risk score and median TMB, LUAD patients were classified into four groups: H-TMB+high-risk, H-TMB+low-risk, L-TMB+high-risk, and L-TMB+low-risk, and a survival curve was generated (**Figure 7E**), which illustrated that the H-TMB+low-risk group has the best prognosis, while the L-TMB+high-risk group has the worst prognosis. The model genes mutation map was displayed in **Figure 7F**, which revealed that the most prevalent type of variant classification in the TCGA-LUAD cohort. Moreover, 14 LUAD patients exhibited mutations, with HDC having the highest mutation frequency. Subsequently, we further explored the co-mutation relationship of the model genes (**Figure 7G**), and the findings indicated that there was no significant co-mutation relationship between most genes, although a weak co-mutation relationship existed between PPP3CA and RGS2, HDC and MYLIP, but it was not statistically significant.

### 3.7 Assessment of immune microenvironment

**Figure 8:**
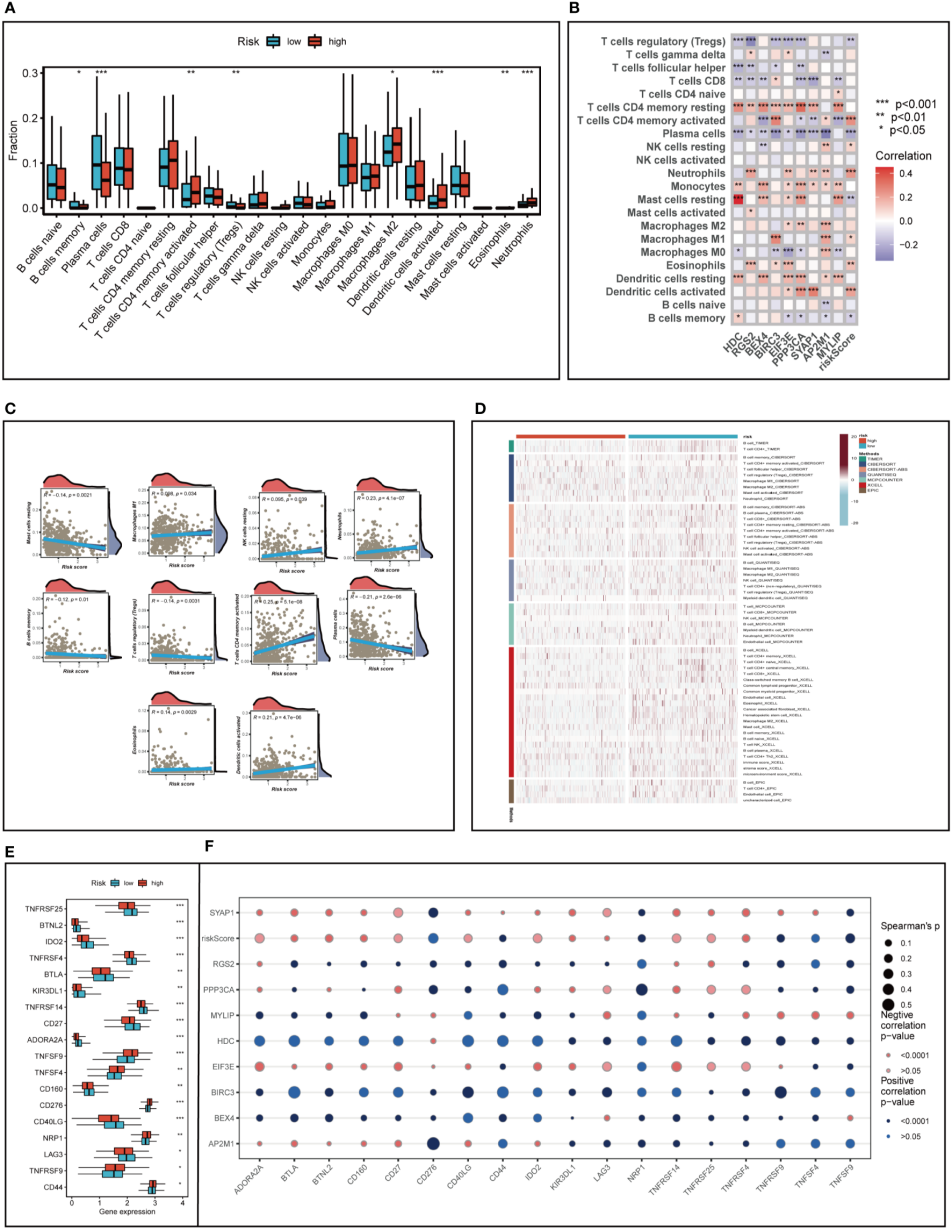
Analysis of immune cell content and immune checkpoint. **(A)** Evaluating the differences in immune cell content between high and low risk groups. **(B, C)** Correlations between immune cell content and model genes and risk scores. **(D)** Differences in immune cell content between risk groups were assessed using seven different algorithms. **(E)** Differences in immune checkpoint gene expression between high and low risk groups. **(F)** Relationships between immunological checkpoints, model genes, and risk scores. *P < 0.05, **P < 0.01, ***P < 0.001.

We analyzed LUAD samples for their immune cell composition using the CIBERSORT method, comparing the high- and low-risk groups and correlating them with model genes and risk scores. As illustrated in **Figures 8A, B** memory cells, plasma cells, and regulatory T cells were relatively low in the high-risk group (*P*<0.05), while memory-activated CD4+T cells, M2 macrophages, activated DC cells, neutrophils, and eosinophils were more prevalent in the high-risk group (*P*<0.05). **Figure 8C** demonstrated that the risk score was negatively associated with the relative content of resting mast cells, plasma cells, memory B cells, and regulatory T cells and positively associated with memory-activated CD4+T cells, neutrophils, activated dendritic cells, M1 macrophages, and resting NK cells. Intriguingly, we discovered that the expression of the HDC gene was significantly correlated with the relative content of resting mast cells, which might suggest that the HDC gene influences TME changes by regulating mast cell proliferation.

**Figure 9:**
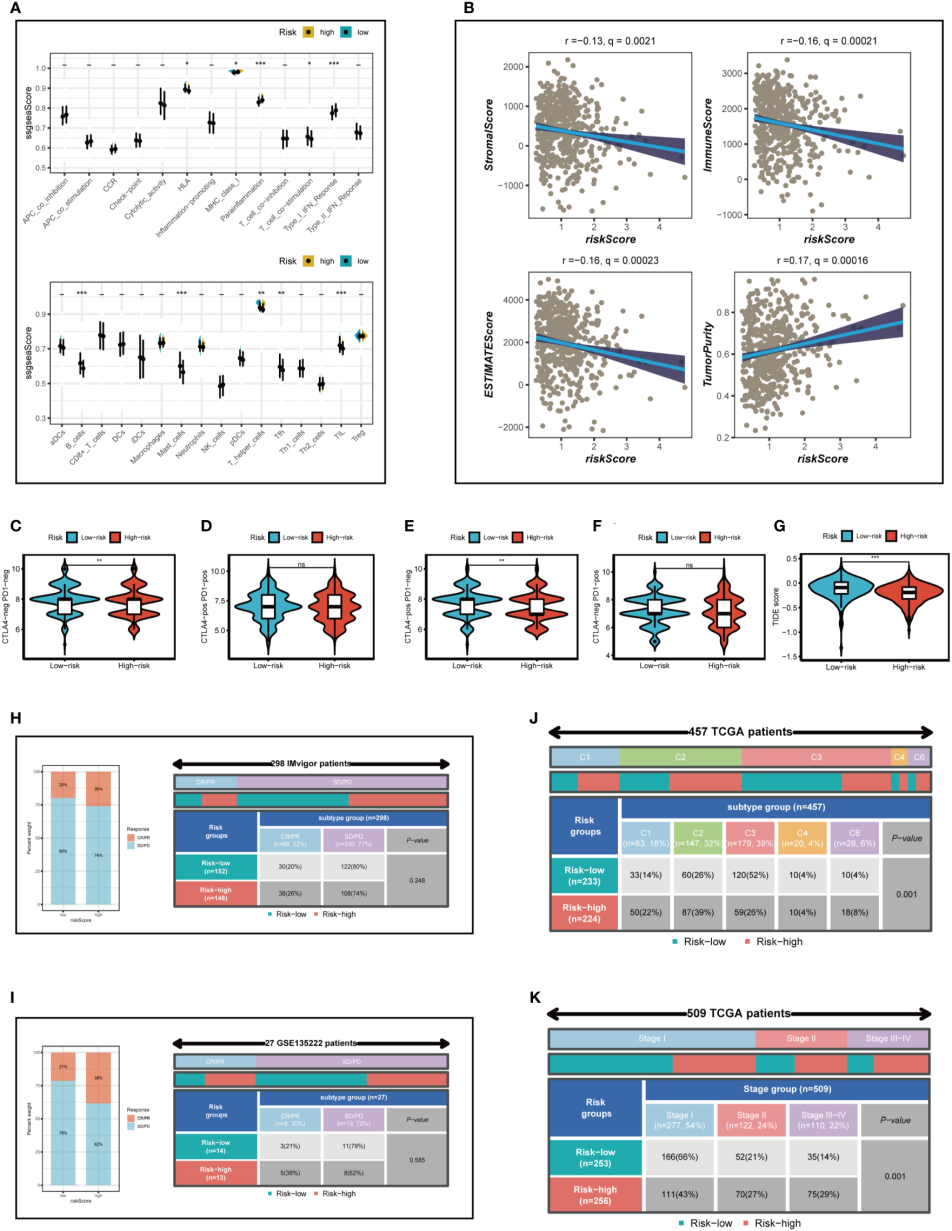
Analysis of immunotherapy and the immune microenvironment. **(A)** The ssgsea algorithm was used to evaluate the differences in immune cells and immune-related functions between high and low risk groups. **(B)** Correlations between risk scores and immune scores, stromal scores, and estimate scores. **(C–G)** Variations in IPS and TIDE between those at high-and low-risk. **(H, I)** The Imvigor210 cohort and GSE135222 independent immunotherapy cohorts were used to verify the benefit of patients in different risk groups from immunotherapy. **(J)** Relationship between high - and low-risk groups and six immune subtypes. **(K)** Correlation between tumor stages and different risk groups. *P < 0.05, **P < 0.01, ***P < 0.001.

To further emphasize the differences in immune infiltration levels between high- and low-risk groups, we examined the results of seven different immune infiltration algorithms. As depicted in **Figure 8D**, the low-risk group exhibited greater immune cell infiltration, including B cells and T cells. ICI therapy has made a significant breakthrough in cancer treatment and is now considered a highly promising immunotherapy approach. Consequently, we assessed the expression levels of immunological checkpoints in various risk groups (**Figure 8E**). In comparison to the high-risk group, the low-risk group exhibited higher expression of most immune checkpoints, including TNFRSF25, BTNL2, IDO2, TNFRSF4, BTLA, KIR3DL1, TNFRSF14, CD27, ADORA2A, CD160, CD40LG, and LAG3. In contrast, TNFSF9, TNFSF4, CD276, NRP1, TNFRSF9, and CD44 were more highly expressed in the high-risk group. Correlations between risk scores, model genes, and immune checkpoints are illustrated in **Figure 8F**. Further analysis of the correlations between differentially expressed immune checkpoint genes, risk scores, and model gene expression revealed a significant positive correlation between risk scores and TNFSF9, suggesting that high-risk patients may benefit from TNFSF9-related immunotherapy. Interestingly, the HDC gene was negatively correlated with most differentially expressed immune checkpoints, indicating that HDC gene expression might predict the efficacy of ICI treatment in lung cancer patients. **Figure 9A** displays the ssgsea results for immune cell and immune-related function enrichment. The distribution of immune cells underscores the distinct tumor immune microenvironments (TIME) in the two LUAD risk groups. Among all immune cell types, mast cells and B cells exhibited higher enrichment in the low-risk group. The low-risk group likely possessed a greater capacity for stimulating the adaptive immune system due to their increased human leukocyte antigen (HLA) activity, potentially explaining the higher OS in this group. Emerging research suggests that mast cells might play a role in modulating the timing and response to immunotherapy (38, 39). To evaluate TIME patterns and immunotherapeutic responses across different risk groups, we performed an analysis using the ESTIMATE method to calculate the components of LUAD’s TIME. Results revealed that risk scores were positively correlated with immune scores, stromal scores, and estimate scores but negatively correlated with tumor purity (**Figure 9B**). Additionally, the Immune Prognostic Score (IPS) could help identify patients who may benefit from immunotherapy. Tumor samples from these patients were expected to demonstrate favorable immune responses to PD-1/PD-L1 or CTLA4 inhibitors, or both (**Figures 9C–F**). Patients in the low-risk group had significantly higher IPS scores when treated with CTLA-4, suggesting that they would benefit the most from this type of immunotherapy.

The TIDE algorithm was used to predict the immunotherapy response of different risk groups. Our findings indicated a significantly lower TIDE score in the high-risk group (*P*<0.001; **Figure 9G**), suggesting that high-risk patients are more likely to benefit from immunotherapy. In conclusion, although the low-risk group displayed higher expression of immune checkpoint genes and greater immune cell infiltration, it also exhibited increased Treg cells, which could lead to an immunosuppressive microenvironment and reduced benefit from immunotherapy. This finding was also confirmed in the subsequent immunotherapy cohort.

### 3.8 Immunotherapy cohort analysis

The Imvigor210 cohort and GSE135222 independent immunotherapy cohorts were employed to validate the advantage of patients in distinct risk groups from immunotherapy. The outcomes revealed that, in accordance with our analysis, the proportion of complete response (CR) and partial response (PR) was elevated in the high-risk group (**Figures 9H, I**), suggesting that patients in the high-risk group were more likely to derive benefit from immunotherapy. Subsequently, the relationship between model categorization and immune subtypes was examined, as depicted in **Figure 9J**. Immune subtypes C1, C2, and C6 were predominantly observed in the high-risk group. Prior research has indicated that the immune subtype C3 group exhibited the most favorable prognosis, and C3 was primarily found in the low-risk category, in line with previous findings. Furthermore, we explored the association between the risk model and the clinical stage of LUAD patients (**Figure 9K**) and uncovered that as the pathological stage advanced, the proportion of high-risk patients increased, thereby demonstrating the reliability of the risk model we established.

### 3.9 Validation of SYAP1 expression and biological function in LUAD

**Figure 10:**
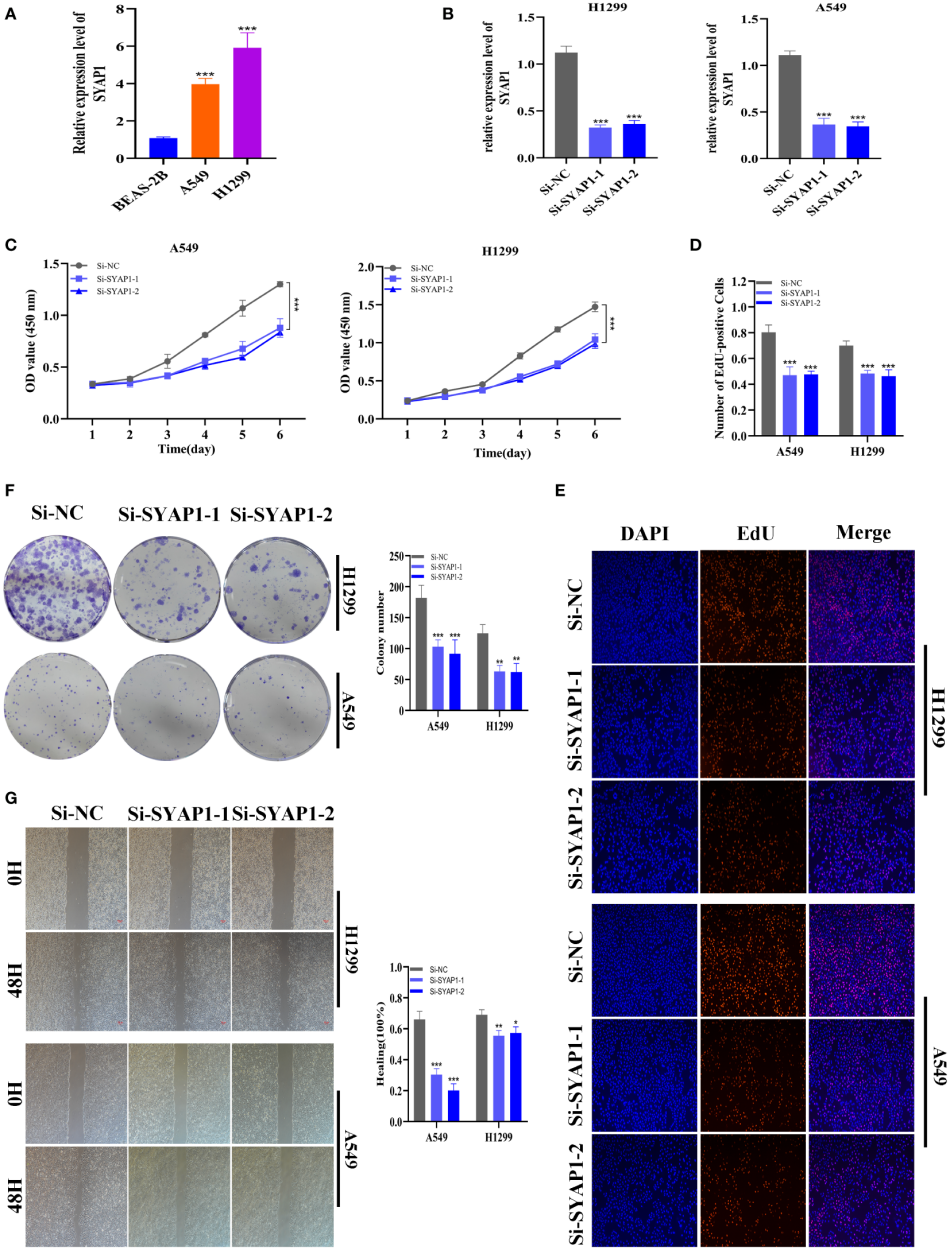
The role of SYAP1 in LUAD. **(A)** SYAP1 was highly expressed in LUAD cell lines compared to healthy human lung epithelial BEAS-2B cell lines. **(B)** RT-qPCR was performed to measure the relative expression of SYAP1 in LUAD cells transfected with si-RNAs or negative control (NC). **(C)** CCK8 assay. After SYAP1 knockdown, the cells showed significant reduction in viability, and si-SYAP1-1 and si-SYAP1-2 had a better knockdown potency, which were used in further in vitro experiments. **(D, E)** EdU staining assay indicated that downregulation of SYAP1 expression repressed cell proliferation in LUAD cell lines. **(F)** Colony formation assay displayed that cell with reduced SYAP1 expression exhibited a significant reduction in the numbers of colonies, compared with the NC group. **(G)** Scratch-wound healing assay. A significantly slower wound healing rate was observed in cells with a decreased expression of the SYAP1 gene. *P < 0.05, **P < 0.01, ***P < 0.001.

**Figure 11:**
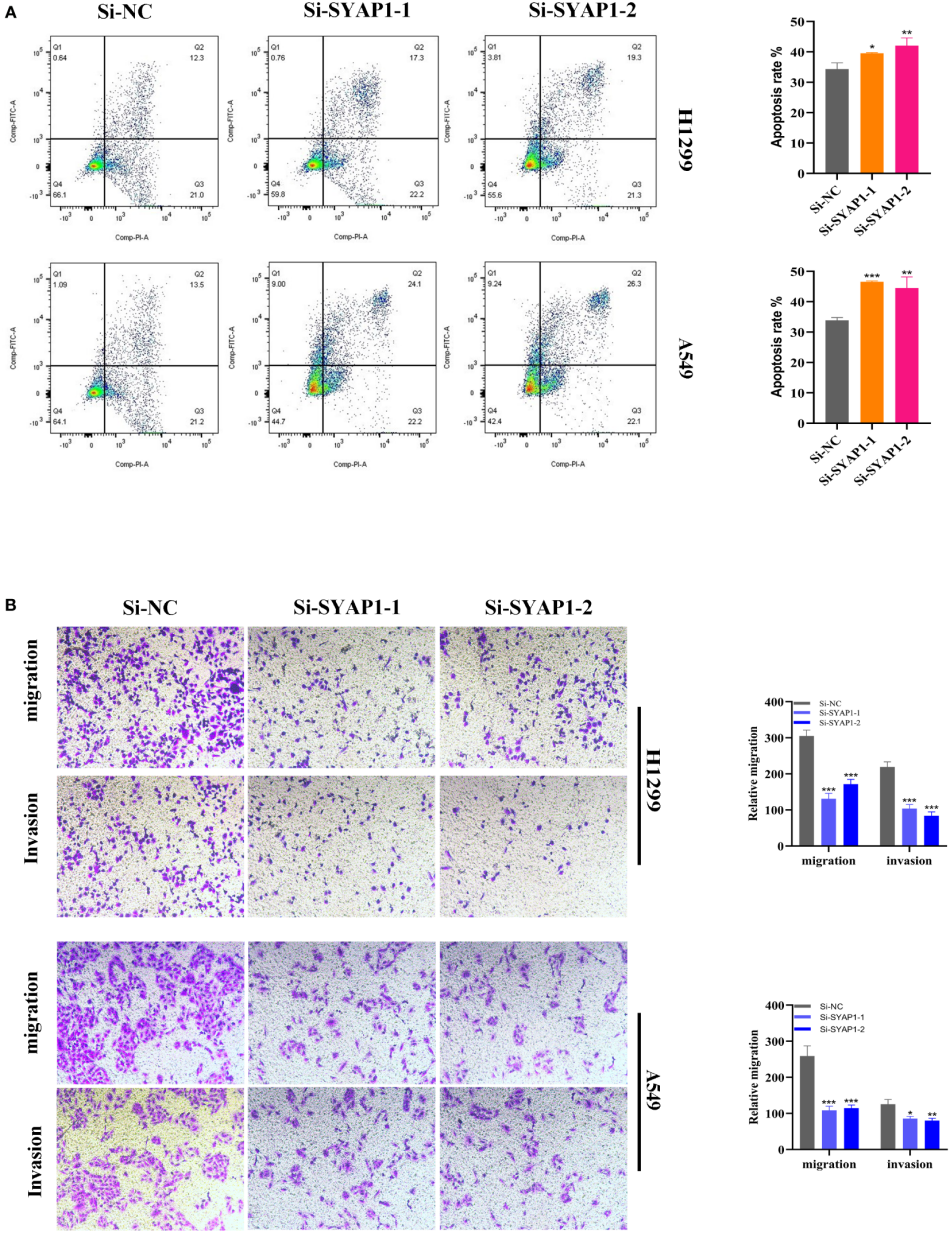
Cell apoptosis and Transwell assay. **(A)** In SYAP1 knockdown LUAD cell lines, apoptosis experiments revealed that the overall number of early and late apoptotic cells was considerably higher than in the NC group. **(B)** Transwell assay showed that downregulation of SYAP1 expression inhibited the migration and invasion capacity of LUAD cells. To demonstrate the accuracy and reproducibility of the results, all experiments were repeated in two LUAD (A549, H1299) cell lines and all data were presented as the means ± SD of three independent experiments. *P < 0.05, **P < 0.01, ***P < 0.001.

Further investigation was conducted on SYAP1, which exhibited the highest hazard ratio (HR) value in the signature. Utilizing the GEPIA database, SYAP1 was found to be significantly expressed in tumor tissues in the majority of malignancies when examining the relative expression of SYAP1 in pan-cancerous tumors and adjacent tumors (**Supplementary Figure 3**) and LUAD patients with high SYAP1 expression have poorer OS (**Supplementary Figure 4**). Prior studies have disclosed that patients with elevated SYAP1 expression in LUAD experienced unfavorable outcomes. To better comprehend SYAP1’s role, we carried out *in vitro* research. **Figure 10A** displays that SYAP1 is markedly elevated in two LUAD (A549, H1299) cell lines compared to BEAS-2B cell lines from healthy human lung epithelial tissue. For subsequent experiments, SYAP1 in LUAD cell lines was silenced. Initially, we assessed SYAP1’s knockdown efficacy in LUAD cells and discovered that, following SYAP1 gene silencing, SYAP1 gene expression in A549 and H1299 cell lines was less than half of the initial level **(** **Figure 10B** **)**. CCK-8 and EdU assays were employed to determine the potential proliferative effect of SYAP1 on LUAD cells. The findings demonstrated that diminished expression of SYAP1 reduced the proliferative capacity of A549 and H1299 cells compared to the control group (**Figures 10C–E**), suggesting that SYAP1 may exert a potentially vital influence on the proliferation of LUAD cell lines. Moreover, the colony formation assay indicated that the downregulation of SYAP1 led to a notable reduction in the number of colonies compared to the control group (**Figure 10F**). The outcome of the scratch-wound healing experiment was analogous. Cells with attenuated SYAP1 gene expression exhibited a considerably decelerated rate of wound healing (**Figure 10G**). The cell apoptosis assay revealed that the apoptosis rate of LUAD cell lines with SYAP1 knockdown was significantly increased (**Figure 11A**). Consequently, it is plausible to hypothesize that overexpression of AYAP1 can impede cell apoptosis and foster tumor progression. Transwell experiments disclosed that SYAP1 downregulation substantially curtailed the migration and invasion of A549 and H1299 cells (**Figure 11B**). To corroborate the precision and reproducibility of the results, all experiments were replicated in two LUAD (A549, H1299) cell lines, and all data were presented as the means ± SD of three independent experiments. *P< 0.05, **P< 0.01, ***P< 0.001.

## 4 Discussion

Previous inquiries delved into the association between immune cell populations and cancer patients’ clinical outcomes (40). Distinct cancer types possess diverse immune cell populations, engendering a complex immunological network within the TME that profoundly influences tumor development and progression. Mast cells, a crucial constituent of the immunological milieu in tumor tissues, may exhibit pro- or anti-tumorigenic functions by releasing various mediators (5). For instance, mast cell-secreted angiogenic and lymphangiogenic factors promote tumor angiogenesis and lymphangiogenesis (41, 42). Numerous matrix metalloproteinases discharged by mast cells impact extracellular matrix degradation in tumors, further facilitating cancer cell distant metastasis (43). Tumor growth in pancreatic cancer is accelerated by MYC activation owing to the swift recruitment of mast cells to the tumor site. MYC governs and directs tissue remodeling, angiogenesis, and inflammation by stimulating mast cells (44). Tryptase AB1 and interleukin-1-beta released by mast cells intensify pleural fluid production and tumor progression by augmenting vascular permeability and activating NF-kB in pleural tumor cells (45). Conversely, mast cells possess the capacity to combat tumors directly via tumor cell cytotoxicity induced by TNF-a and ROS or indirectly through the synthesis of interleukin-9 and heparin and the initiation of dendritic cell maturation (5). Mast cells’ multifaceted capabilities enable them to execute diverse functions across various cancer subtypes and stages.

Several conflicting studies have reported the impact of mast cells on LUAD patient outcomes. One investigation posited that stage I LUAD patients with mast cells experienced unfavorable outcomes due to angiogenesis (43). Another study discerned that KIT-competent mast cells contribute to LUAD development by producing interleukin-1b, which propels the formation, proliferation, and metastasis of KRAS-mutant LUAD (46). Conversely, one investigation demonstrated that only mast cells were associated with a better prognosis in LUAD according to univariate analysis (47). Mast cell infiltration is more prevalent in low-grade histologic subtypes than in high-grade subtypes, as per a prior study, although the findings of these investigations exhibit considerable variability concerning mast cell prognostic significance (48). Comprehending the diverse mechanisms and activities of mast cells in LUAD may prove valuable in devising immunotherapy. Employing single-cell analysis, specific mast cell populations in LUAD were identified, and the differential genes were appraised as mast cell marker genes for future investigation. A mast cell-related risk score was computed based on the expression levels of the mast cell-related gene signature using Cox and lasso regression. LUAD patients were stratified into high- and low-risk groups according to the median risk score, and differences in OS, enrichment pathways, mutation information, immune infiltration, the TME, and responsiveness to immunotherapy were examined. Two independent GEO cohorts were utilized to validate the mast cell-related signature. To substantiate our analytical findings, the gene exhibiting the highest HR value among the model genes was selected for the cell function experiment.

Patients in the high-risk category exhibited a diminished life expectancy. To elucidate this phenomenon, we compared mutation information between high- and low-risk groups, uncovering a heightened TMB in high-risk patients. Intriguingly, our study disclosed that patients with elevated TMB demonstrated superior OS, counter to our initial findings. We further investigated the pathway enrichment of high- and low-risk groups to comprehend the reasoning. Remarkably, the GSVA revealed enrichments in mTORC1 signaling and c-MYC-associated pathways within the high-risk group. Prior research has established that mTORC1 signaling fosters cancer progression (49–51). Mast cells assume pivotal roles in MYC activation and may promote tumor growth in pancreatic cancer (44). We can surmise that in LUAD’s TME, mast cells may activate MYC to facilitate tumorigenesis and advancement.

According to recent studies, genetic alterations are directly correlated with neoantigen formation and immunotherapeutic responses (52). However, our findings indicate that low-risk patients have reduced TMB, and high-frequency mutated genes manifest more frequently in the high-risk group, suggesting that high-risk patients may be more receptive to immunotherapy. Subsequent analysis supports this notion.

We meticulously evaluated patients’ responses to immunotherapy. TIDE algorithms were employed to estimate lung cancer patients’ potential to benefit from immunotherapy. Patients in the low-risk group exhibited higher TIDE scores, signifying enhanced sensitivity to anti-PD1 treatment for those with high-risk scores, corroborating our previous findings. Moreover, we examined two external immunotherapy cohorts and computed risk scores for each patient, determining that individuals in the high-risk category were more likely to profit from immunotherapy. High-risk patients derive benefits from both chemotherapy and immunotherapy. In the ensuing immune cell correlation analysis, we discerned a significant negative association between the risk score and Treg cells, indicating the low-risk group’s higher proportion of Treg cells, which engendered an immunosuppressive TME, further explaining why the high-risk group was more prone to benefit from immunotherapy.

To substantiate the efficacy of our prognostic model, we evaluated its precision using ROC and tested it in two external independent cohorts. The results confirmed the stability of our 9-gene model. Additionally, we used two external immunotherapy cohorts to verify that high-risk patients were more likely to benefit from immunotherapy. Intriguingly, we found that SYAP1 had the highest hazard ratio among the nine modeled genes, and further survival analysis revealed that elevated SYAP1 expression was associated with poor prognosis in LUAD patients. Subsequently, we conducted cell function experiments on two LUAD cell lines, A549 and H1299, which showed that silencing SYAP1 significantly attenuated cell proliferation, invasion, migration, and promoted apoptosis.

In conclusion, we developed a reliable prognostic model for predicting the prognosis and responsiveness to immunotherapy in LUAD patients. Our findings suggest that SYAP1 may represent a novel therapeutic target for individuals with LUAD.

## Data availability statement

The original contributions presented in the study are included in the article/**Supplementary Material**. Further inquiries can be directed to the corresponding authors.

## Author contributions

PZ, JLL and SP contributed conception and design of the study, JLL and JHL collected the data; PZ performed the statistical analysis; PZ wrote the first draft of the manuscript; JL and JHL revised the manuscript; JX and JHL gave the final approval of the version to be submitted. All authors contributed to manuscript and approved the submitted version.
