# Correction: Mast cell marker gene signature: prognosis and immunotherapy response prediction in lung adenocarcinoma through integrated scRNA-seq and bulk RNA-seq

**DOI:** 10.3389/fimmu.2026.1827500

**Published:** 2026-03-27

**Authors:** Pengpeng Zhang, Jianlan Liu, Shengbin Pei, Dan Wu, Jiaheng Xie, Jinhui Liu, Jun Li

**Affiliations:** 1 Department of Thoracic Surgery, The First Affiliated Hospital of Nanjing Medical University, Nanjing, China; 2 Department of Burns and Plastic Surgery, The First Affiliated Hospital of Nanjing Medical University, Nanjing, China; 3 Department of Breast Surgery, The First Affiliated Hospital of Nanjing Medical University, Nanjing, China; 4 Department of Rheumatology and Immunology, The Affiliated Drum Tower Hospital of Nanjing University Medical School, Nanjing, China; 5 Department of Gynecology, The First Affiliated Hospital of Nanjing Medical University, Nanjing, China

**Keywords:** lung adenocarcinoma, signature, mast cell, tumor immune microenvironment, SYAP1

In the original article, there was an error in Section 2.2 “*Processing flow of scRNA-seq data*”.

Original text:

“Cells with “min.cells<3” and “min.features>200” were excluded. After filtering out cells with nFeature RNA > 7000 and > 10% mitochondrial sequencing count, a total of 46286 cells were retained for further analysis”.

Should be corrected to:

“Genes expressed in fewer than 3 cells (min.cells = 3) and cells with fewer than 200 detected features (min.features = 200) were excluded. After filtering out cells with nFeature RNA > 7000 and mitochondrial sequencing count > 10%, a total of 46286 cells were retained for further analysis”.

We confirm that the actual data processing was performed correctly using the appropriate parameter settings. This error was purely a descriptive inaccuracy and does not affect any of the results, figures, conclusions, or interpretations presented in the article.

The original version of this article has been updated.

