# Supplementary material for: Mast cell marker gene signature: prognosis and immunotherapy response prediction in lung adenocarcinoma through integrated scRNA-seq and bulk RNA-seq
Source: Front Immunol. 2023 May 15;14:1189520. doi: 10.3389/fimmu.2023.1189520 (PMC10225553; doi:10.3389/fimmu.2023.1189520)
Supplement: Supplementary file 1 [file DataSheet_1.docx]

Supplementary Material

# Supplementary Figures


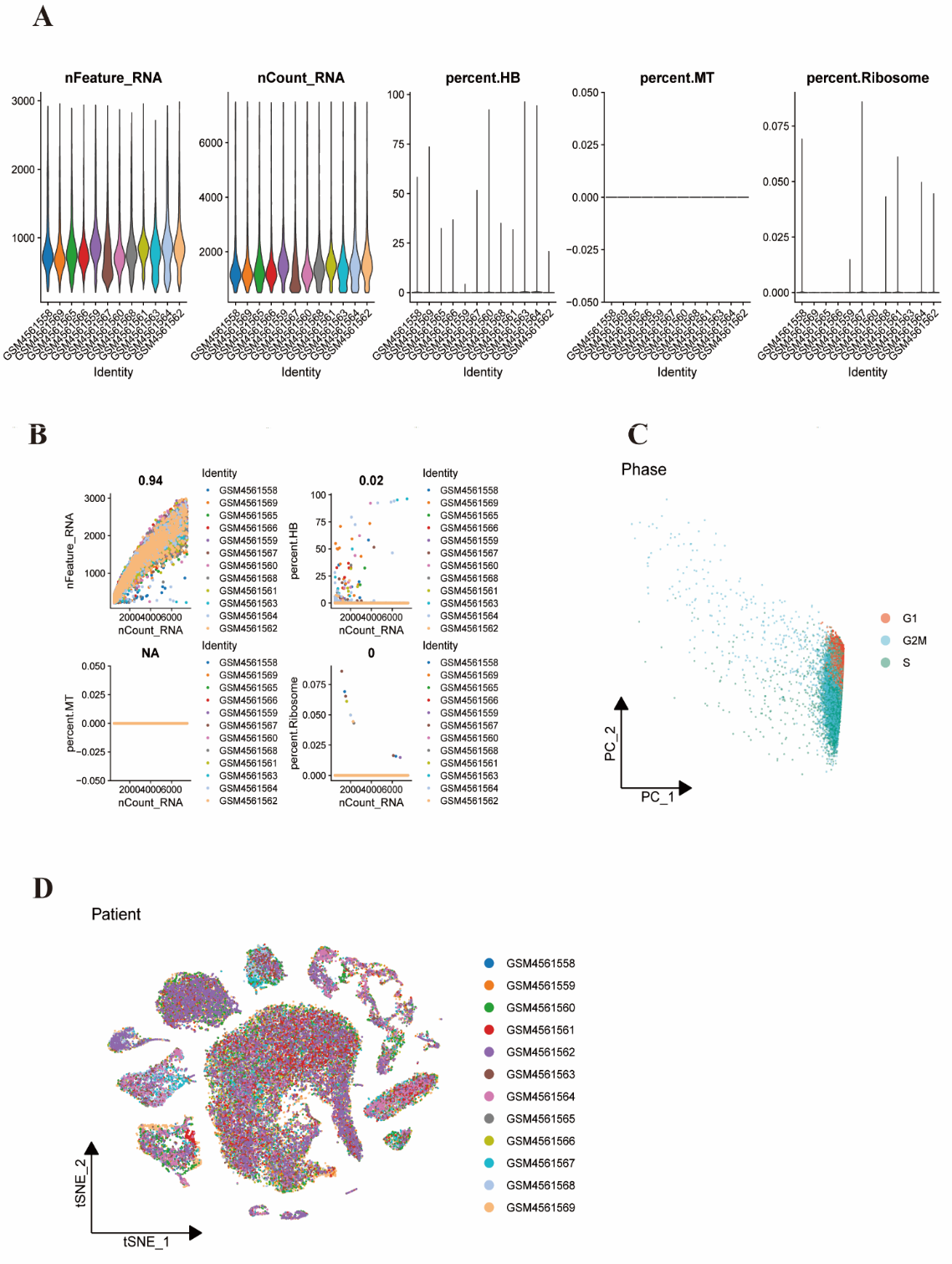


**Supplementary Figure 1.** Quality control of single-cell sequencing data. (A) The distribution of gene expression levels, sequencing depth, the percentage of red blood cell genes, the percentage of mitochondrial genes and the percentage of ribosome genes in the 12 samples. (B) Correlation between sequencing depth and gene expression levels, the percentage of mitochondrial genes, the percentage of red blood cell genes, the percentage of ribosome genes. (C) Cell cycle related genes were used for dimensionality reduction clustering. (D) The cell distribution of the 12 samples was shown by tsne.


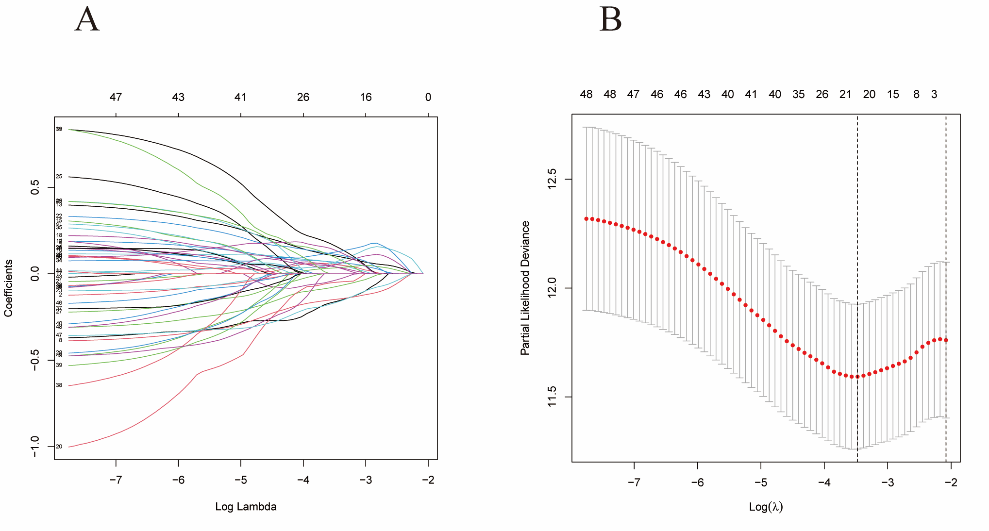


**Supplementary Figure 2.** Construction and validation of MRGs prognostic model. (A, B) Twenty genes were selected for multivariate regression analysis using Lasso regression.


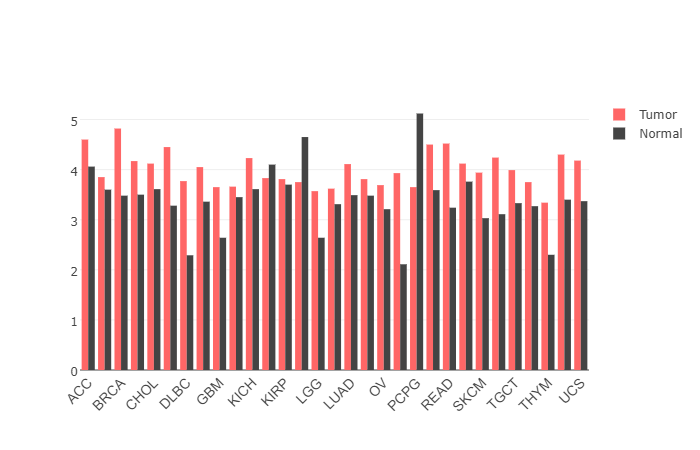
 **A**

**Supplementary Figure 3.** Expression of SYAP1 in pan-carcinoma (A) Numerous malignancies express SYAP1 differently, and lung cancer exhibits notable variations as well.


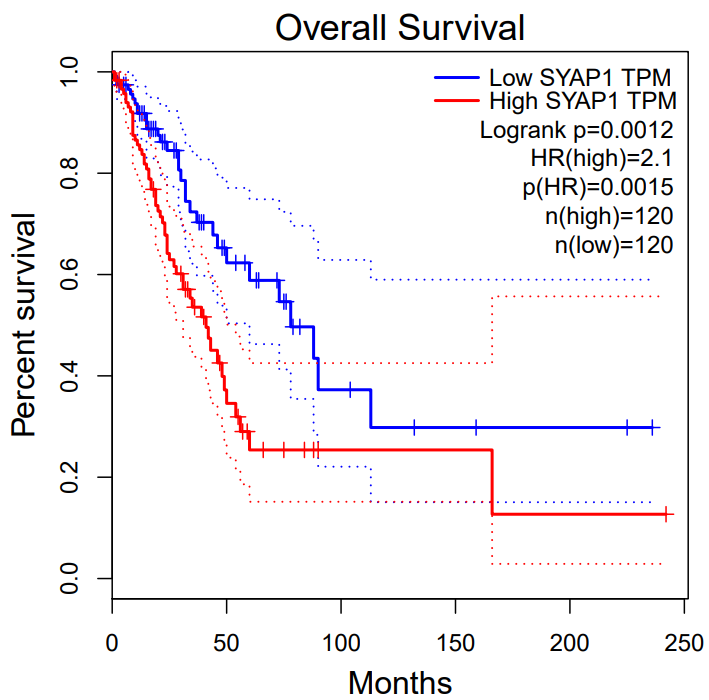


**Supplementary Figure 4.** The prognostic effect of SYAP1 on survival was verified using the GEPIA database, which showed that lung adenocarcinoma patients with high expression of SYAP1 had poor survival.
