# Supplementary material for: Mast cell marker gene signature: prognosis and immunotherapy response prediction in lung adenocarcinoma through integrated scRNA-seq and bulk RNA-seq
Source: Front Immunol. 2023 May 15;14:1189520. doi: 10.3389/fimmu.2023.1189520 (PMC10225553; doi:10.3389/fimmu.2023.1189520)
Supplement: Supplementary file 2 [file Table_1.docx]

| **Oligonucleotides** | **Nucleotide sequence (5'-3')** |
| --- | --- |
| **siRNA** |  |
| siRNA-NC | GCUUCGCGCCGUAGUCUUA |
| si SYAP1-1 | GCTGCTAAGCAAGATGAGATT |
| si SYAP1-2 | CCTTCGATGCCTGTAACCTAA |
| **Primer** |  |
| GAPDH | GGCCTCCAAGGAGTAAGACC (forward) |
|  | AGGGGAGATTCAGTGTGGTG (reverse) |
| SYAP1 | GCCTTCGATGCCTGTAACCT (forward) |
|  | TTCCAGTACGGCTGTCTCCT (reverse) |
|  |  |

**Supplementary Table S1. Oligonucleotides used in research**
